# Prognostic Implication of Histological Oligodendroglial Tumor Component: Clinicopathological Analysis of 111 Cases of Malignant Gliomas

**DOI:** 10.1371/journal.pone.0041669

**Published:** 2012-07-24

**Authors:** Hiromi Kanno, Hiroshi Nishihara, Takuhito Narita, Shigeru Yamaguchi, Hiroyuki Kobayashi, Mishie Tanino, Taichi Kimura, Shunsuke Terasaka, Shinya Tanaka

**Affiliations:** 1 Laboratory of Cancer Research, Department of Pathology, Hokkaido University School of Medicine, Sapporo, Japan; 2 Laboratory of Translational Pathology, Hokkaido University School of Medicine, Sapporo, Japan; 3 Department of Neurosurgery, Hokkaido University School of Medicine, Sapporo, Japan; University of Tokyo, Japan

## Abstract

The favorable prognosis of high-grade oligodendroglial tumor such as glioblastoma (GBM) with oligodendroglioma component (GBMO) has been suggested; however, the studies which examine the prognostic significance of oligodendroglial tumor were limited. In this study, we performed a histopathology-based reevaluation of 111 cases of high grade gliomas according to the latest World Health Organization (WHO), and compared the clinical outcomes between oligodendroglial tumors and pure astrocytic tumors. The survival analysis revealed that the patients with high grade oligodendroglial tumor including GBMO significantly indicated better prognosis compared to the patients with high grade pure astrocytic tumors (GBM and AA, anaplastic astrocytoma) as expected, and the obtained survival curves were almost identical to those from the patients with conventional Grade III or Grade IV tumors, respectively. Moreover, if the cases of oligodendroglial tumor were histopathologically excluded, the patients with AA exhibited extremely poor prognosis which was similar to that of GBM, suggesting that the histological identification of oligodendroglial tumor component, even partially, prescribe the prognosis of high grade glioma patients. This is the prominent report of retrospective clinicopathological analysis for high-grade gliomas throughout Grade III and IV, especially referring to the prognostic value of histological oligodendroglial tumor component; in addition, our results might offer an alternative aspect for the grading of high-grade astrocytic/oligodendroglial tumors.

## Introduction

High grade gliomas/malignant gliomas are composed of astrocytic and/or oligodendroglial tumors which are categorized into WHO grade III and IV. The clinical outcome of the patients with these diseases remains extremely poor, although the oligodendroglial tumors are reported to exhibit relatively favorable prognosis compared to the astrocytic tumors [1], [2], [3]. In addition to variable prognostic factors such as the age of the patients, the extent of resection or postoperative radiation therapy, tumor grade and Karnofsky performance status (KPS) score, the presence of the oligodendroglial tumor component, prominent microvascular proliferation and/or necrosis in high-grade glioma are focused upon in the recent edition of WHO Classification (4^th^, 2007) [4]. “Glioblastoma with oligodendroglioma component” was placed into Grade IV, and anaplastic oligoastrocytoma (AOA) with microscopical necrosis, formerly categorized in Grade III, is also regarded as Grade IV. In recent reports, the survival analysis of Glioblastoma (GBM) vs. Glioblastoma with oligodendroglioma component (GBMO) was performed and showed no significance between them [5], [6], although some other reports have indicated a better prognosis for GBMO [7], [8], [9], [10]. In addition, a detailed survival analysis limited in Grade III gliomas, between oligodendroglial tumor (AO, AOA) and pure astrocytic tumor (Anaplastic astrocytoma, AA), has not been reported, especially after the recent edition of WHO Classification; therefore, the clinicopathological significance of the oligodendroglial tumor component is still controversial.

Here we reviewed and analyzed 111 cases of high grade gliomas based on the latest WHO classification, and found the critical implication between the prognosis and histological evaluation, especially the presence of the oligodendroglial tumor component.

**Table 1:** Characteristics of 111 Patients.

Characteristics	Number of patients (%)
Median age (range)	57 (11–83)
Gender	
Male	64 (57.7)
Female	47 (42.3)
Extent of surgery	
Biopsy	22 (19.9)
Partial resection	26 (23.4)
Subtotal resection	21 (18.9)
Gross total resection	40 (36.0)
No data	2 (1.8)
Chemotherapy	
ACNU	50 (45.0)
TMZ	40 (36.0)
CDDP	2 (1.8)
CBDCA	1 (0.9)
None	17 (15.3)
No data	1 (0.9)
Radiation therapy	
Yes	101 (91.0)
No	9 (8.1)
No data	1 (0.9)
Preoperative KPS score	
80≧	72 (64.9)
80<	35 (31.5)
No data	4 (3.6)

ACNU: Nimustine hydrochloride, TMZ: Temozolomide, CDDP: Cisplatin, CBDCA: Carboplatin, KPS: Karnofsky performance status.

## Materials and Methods

### Patients

This study was performed with the approval of the Internal Review Board on ethical issues of Hokkaido University Hospital and Graduate School of Medicine, Sapporo, Japan. The samples and the patients’ information were obtained under a blanket written informed consent. Among the patients who were treated at the department of neurosurgery of Hokkaido University Hospital or its affiliated hospitals between 2000 and 2009, we had 133 cases of malignant gliomas (AA, AO, AOA, GBM and GBMO). We performed immunohistochemistry with anti-Olig2 and Glial fibrillary acidic protein (GFAP) antibodies during the initial diagnosis process for confirmation of glial tumor, and we observed all of the 111 case were positive for at least one of these two antibodies. The clinical outcomes of these patients were collected retrospectively. Among these patients, 22 cases were excluded because of following reasons; 13 patients had clinical or histopathological evidence of preceding low grade glioma, 7 patients’ pathological diagnosis couldn’t reach the final consensus and 2 patients’ survival time wasn’t available. The seven patients whose diagnoses couldn’t be determined includes 4 cases with insufficient tissue volume, 1 case suspected as oligoastrocytoma, WHO Grade I, and 2 cases suspected as Primitive neuroectodermal tumor (PNET). There was no case which needs to be differentiated from metastatic carcinoma or other intracranial tumor. Finally, 111 cases were applied for the overall survival analysis. Among these 111 cases, the data of progression-free survival was available in 78 patients.

**Table 2:** Summary of the cases of altered diagnosis.

Initial diagnosis	Altered diagnosis	Number of patients
AA (14)	AO	1
	AOA	2
AO (9)	AOA	3
AOA (20)	AO	4
	GBMO	4
GBM (68)	AOA	1
	AA	2
	GBMO	13
Total (111)		30

AA: anaplastic astrocytoma, AO: anaplastic oligodendroglioma, AOA: anaplastic oligoastrocytoma, GBM: glioblastoma, GBMO: glioblastoma with oligodendroglioma component.

### Histopathological Studies

Here we performed this analysis as a retrospective study. Pathological review of all surgical or biopsy specimens were performed by four pathologists (S.T, H.N, M.T and H.K) who were blind to the clinical information. Routinely formalin-fixed, Paraffin-embedded tissue sections of tumor were stained with Hematoxylin and eosin (H&E) and used for pathological review. Histological features including cellularity, cellular atypia, mitotic activity, necrosis, and microvascular proliferation were reevaluated and diagnosis was made according to the 2007 WHO classification. The oligodendroglial tumor component, such as the oligodendroglioma component in GBM, was defined as the presence of at least 5 tumor cells with obvious perinuclear halo in cluster or even in diffuse, scattered pattern in high power field of H&E section, and we did not refer to any immunohistochemical staining such as Olig-2 or GFAP. The cells which had round nuclei or a microcystic pattern without a perinuclear halo were not regarded as an oligodendroglial tumor component.

**Table 3:** Final diagnosis of the 111 cases.

Histological subtype	Number of patients (%)
AO	11 (10)
AOA	18 (16)
GBMO	17 (15)
AA	13 (12)
GBM	52 (47)
**Total**	**111**

AA: anaplastic astrocytoma, AO: anaplastic oligodendroglioma,

AOA: anaplastic oligoastrocytoma, GBM: glioblastoma,

GBMO: glioblastoma with oligodendroglioma component.

### FISH (Fluorescence in situ Hybridization) Analysis

Among the patients who underwent surgery between 2006 and 2009, eighteen samples were available for FISH analysis to detect the chromosome 1 (1p) deletion. The analysis was conducted using paraffin embedded tissue as previously described [11]. The fluorochrome-labeled probes mapping to 1p36 was used for the detection of 1p loss. Approximately 100 nonoverlapping nuclei were enumerated per hybridization. The deletion for 1p was defined as more than 30% of tumor nuclei containing 1 signal for 1p36.

**Figure 1:**
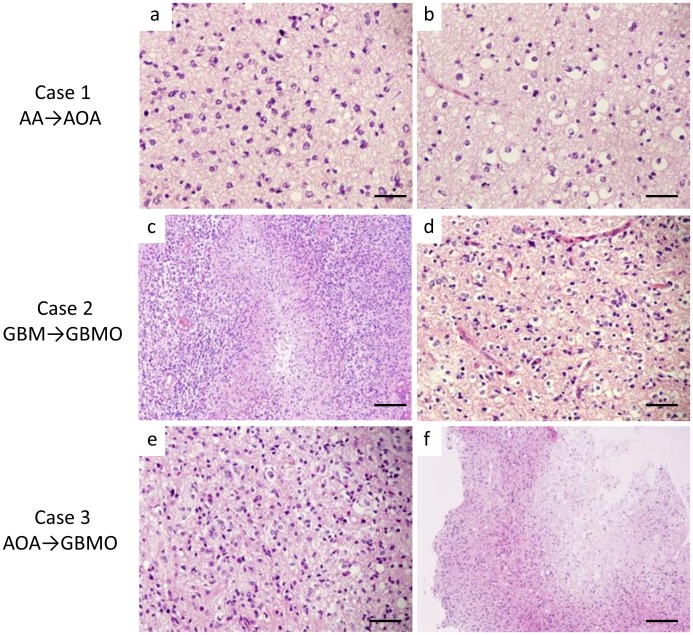
The histological appearance of the cases in which the diagnosis was altered. Case 1: The initial diagnosis was AA because of the prominent atypical astrocytic cells (a). After the histological review, an obvious oligodendroglial tumor component with perinuclear halo was identified (b), and the histological diagnosis was altered to AOA. Case 2: Dense infiltrate of atypical large tumor cells with necrosis indicate GBM (c). However, we found the oligodendroglioma component within the section (d), thereby changing the final diagnosis to GBMO according to the 2007 WHO classification. Case 3: The tumor consists of middle-sized atypical astrocytic cells with eosinophilic cytoplasm and also atypical oligodendroglial cells with perinuclear halo, giving the initial diagnosis as AOA (e). The cellularity and nuclear atypia of this case is moderate; however, the presence of micronecrosis (f) in this lesion enforced us to alter the diagnosis to GBMO. (The scale bars represent 50 µm (a, b, d, and e) and 100 µm (c, f).).

### Statistical Analysis

Time to progression and survival, measured from the date of first surgical resection or biopsy to disease progression and death, respectively, or the date of last follow-up visit was analyzed by the Kaplan-Meier method. Log-rank test was employed for comparing the curves.

**Figure 2:**
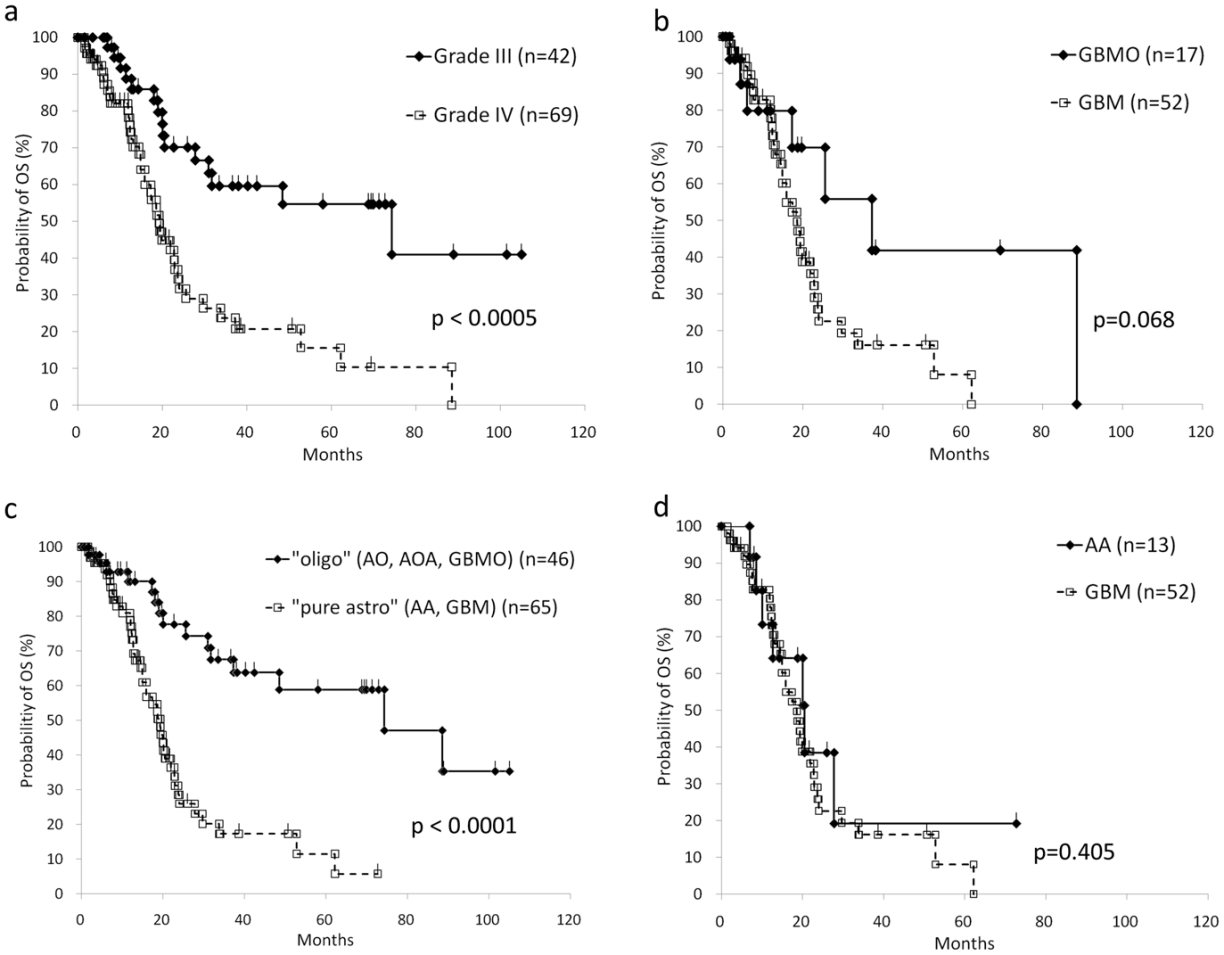
Overall survival (OS) analysis based on the histological subclassifications. a: Conventional Grade III gliomas (AA, AO and AOA) show significantly better prognosis than Grade IV gliomas (GBM, GBMO). b: GBMO presented longer survival compared to GBM, although it is statistically not significant (p = 0.068). c: Oligodendroglial tumor (“oligo”; AO, AOA, GBMO) shows significantly better prognosis compared to pure astrocytic tumor (“pure astro”; AA, GBM). d: The survival curve of AA patients is almost identical to that of GBM patients.

## Results

### Characteristics of Patients

The summary of included patients is shown in Table 1. Median age of the patients was 57 years (ranging from 11 to 83). Sixty-four patients were men and 47 were women. The treatments included surgical resection, adjuvant radiation and adjuvant chemotherapy. Twenty-two patients (19.9%) underwent biopsy, 26 patients (23.4%) underwent partial resection, 21 patients (18.9%) underwent subtotal resection and 40 patients (36.0%) underwent gross total resection, while the details of 2 patients (1.8%) were unknown. Among the 111 patients, 101 patients (91.0%) received radiation therapy: basically the patients with Grade III glioma received 54 Gy/27 fr, and patients with Grade IV glioma received 60 Gy/30 fr. Chemotherapy was applied to 50 patients (45.0%) with nimustine hydrochloride (ACNU), and 40 patients (36.0%) with temozolomide (TMZ), 2 patients (1.8%) with cisplatin (CDDP), and 1 patient (0.9%) with carboplatin (CBDCA), while 17 patients (15.3%) didn’t receive any chemotherapy. The preoperative KPS score of 72 patients (64.9%) was more than 80 and that of 35 patients (31.5%) was lower than 80. After the treatments, the patients were followed in the outpatient clinic until either their death or their last visit. The mean duration of the follow-up was 24.3 months (range, 0.7–105.0). The detailed information of 13 cases of AAs was as follows. Median age of the patients was 48.2 years (ranging from 15 to 68). Seven patients were men and 6 were women. Seven patients (53.8%) underwent biopsy, 3 patients (23.1%) underwent partial resection, 1 patient (7.7%) underwent subtotal resection and 2 patients (15.4%) underwent gross total resection. Among the 13 patients, 12 patients (92.3%) received radiation therapy. Chemotherapy was applied to all patients; 9 (69.2%) with nimustine hydrochloride (ACNU), and 4 patients (30.8%) with temozolomide (TMZ). The mean duration of the follow-up was 19.5 months (range, 6.9–72.7).

**Figure 3:**
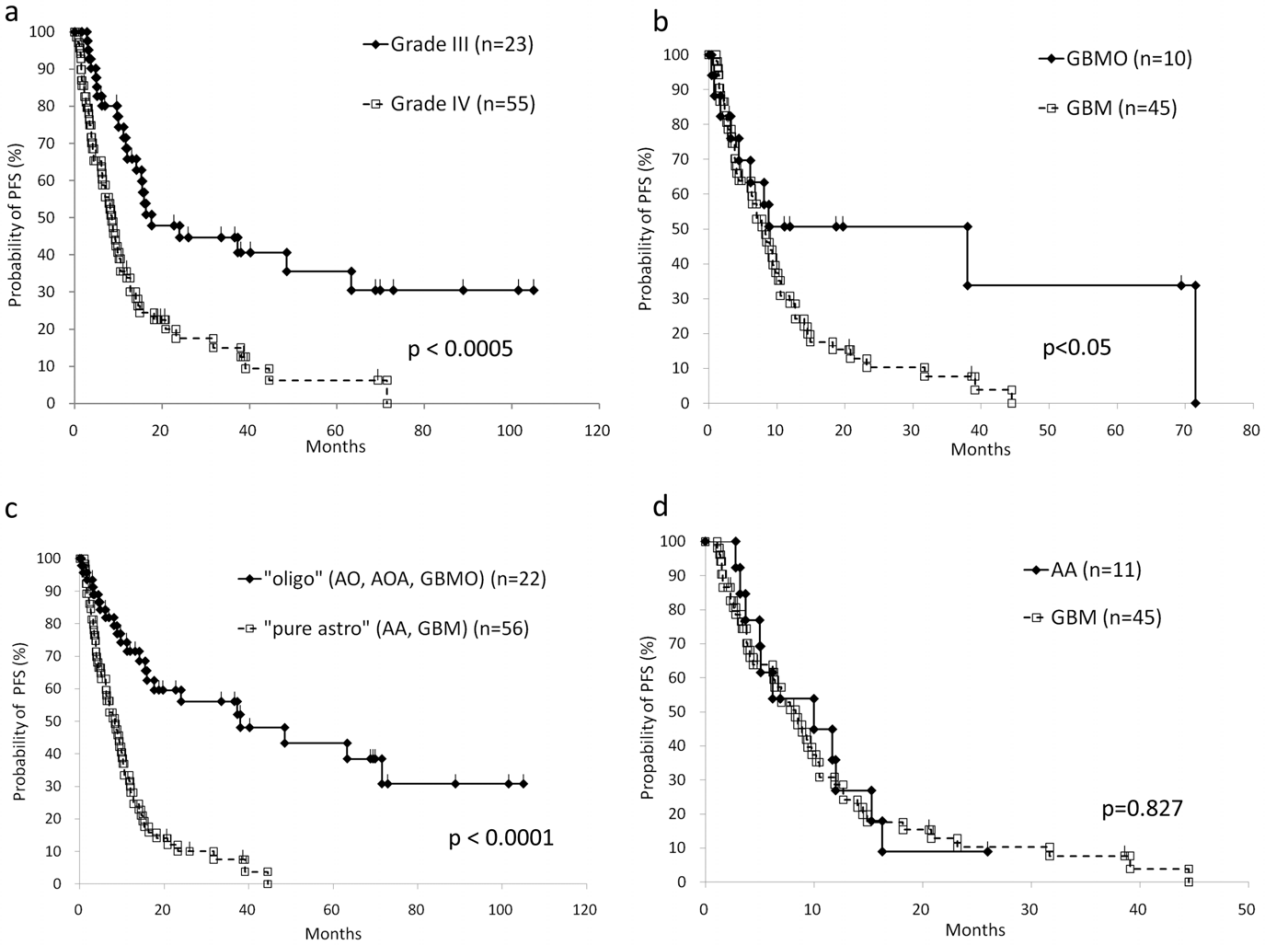
Progression-free survival (PFS) analysis based on the histological subclassifications. a: Conventional Grade III gliomas (AA, AO, AOA) show significantly longer PFS than Grade IV gliomas (GBM, GBMO). b: A significantly longer PFS in GBMO patients was observed compared to GBM (p = 0.0456), while the OS was not significant (Fig. 2b). c: Oligodendroglial tumor (“oligo”; AO, AOA, GBMO) shows significantly longer PFS compared to pure astrocytic tumor (“pure astro”; AA, GBM). d: The PFS curve of AA patients is almost identical to that of GBM patients.

### Histological Evaluation

Because the 111 studied cases of grade III and IV malignant gliomas included the cases diagnosed before 2007, we first performed a histological review of all 111 cases based on the recent edition of WHO Classification (4th, 2007) [4] to obtain the unified pathological diagnosis. As summarized in Table 2, the initial diagnosis of 30 cases of malignant glioma was altered: 17 cases of newly established GBMO were included, and 3 cases of AA were re-categorized into AO or AOA because of the presence of an obvious oligodendroglial lesion, resulting in the additional 17 cases of oligodendroglial tumors (AO, AOA and GBMO). We identified micronecrosis in the 4 cases of AOA; thus their diagnosis was altered to GBMO. The final pathological diagnosis after the review is summarized in Table 3. The histological appearance of the cases in which the pathological diagnosis was altered is exhibited in Fig. 1, while the typical histological appearances of high-grade gliomas (AA, AO, AOA and GBM) are shown in Fig. S1.

**Figure 4:**
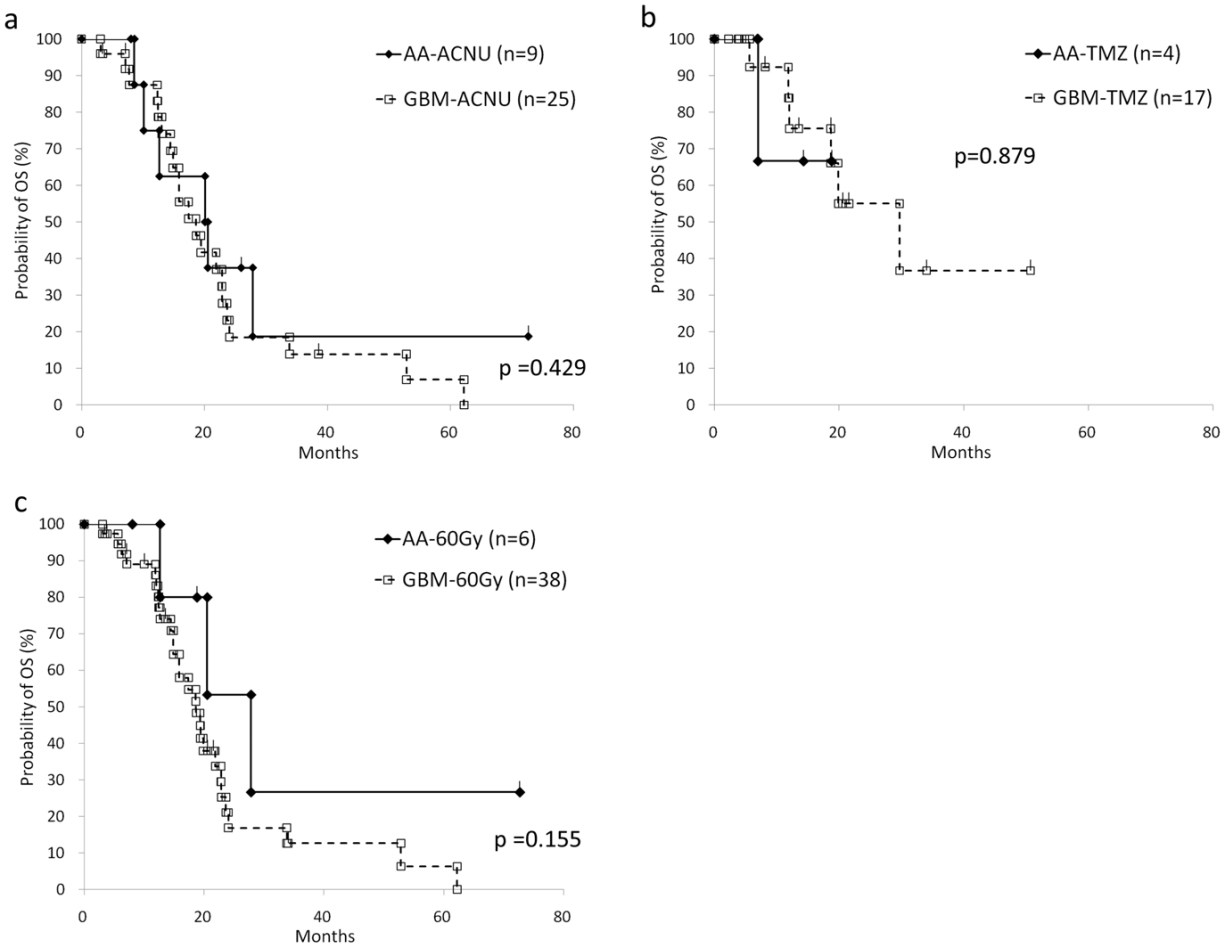
Overall survival analysis of AA and GBM according to the treatment variations. The graph shows comparison of OS between AA and GBM patients who underwent Nimustine hydrochloride (ACNU) - based chemotherapy (a), Temozolomide (TMZ) - based chemotherapy (b), and 60 Gy of radiation therapy (c). There is no statistical siginificance.

### Survival Analysis Based on the Reviewed Pathological Diagnosis

The overall survival (OS) based on the conventional grading entities of gliomas, i.e. Grade III (AO, AOA and AA) versus Grade IV (GBM and GBMO) by the Kaplan-Meier method is shown in Fig. 2a, and is approximately similar to that described in previous reports [12], [13]. Between GBM and GBMO, the statistical significance of OS was not obtained, although the progression-free survival (PFS) of GBMO was statistically better than that of GBM (p = 0.0456) (Fig. 2b and 3b). To clarify the prognostic value of the presence of oligodendroglial tumor component, we divided all cases into two groups regardless of WHO grading, i.e., oligodendroglial tumor (AO, AOA, GBMO) and pure astrocytic tumor (AA, GBM), and obtained the interesting result that the both OS and PFS of oligodendroglial tumor were significantly better than these of pure astrocytic tumor (Fig. 2c and 3c). Furthermore, we found the striking data between AA and GBM; their survival curves of the OS and the PFS were almost identical (Fig. 2d, 3d and S3). In our facility, the patients with pathological Grade III glioma (AA, AO and AOA) were treated with a smaller amount of radiation (54 Gy) compared to the patients with Grade IV (GBM and GBMO; 60 Gy), and the selection of chemotherapy varied according to the standard protocol of the time of onset. To exclude the possible effects due to the variation of chemotherapy and the total amount of irradiation, we analyzed the OS between AA and GBM with ACNU, TMZ, or 60 Gy of irradiation, respectively, and confirmed that the OS and PFS were not affected by the variation of the treatment (Fig. 4).

### FISH Analysis

Among 18 specimens obtained between 2006 and 2009, nine cases were not applied because of the poor preservation status and/or the shortage of the specimens. Finally, we could detect the signal of fluorescent probe in 9 cases, which include 3 oligodendroglial tumors (3 AOAs) and 6 pure astrocytic tumors (1 AA and 5 GBMs) (Table S1). As a result, four cases showed high percentages (higher than 30%) of 1p36 loss and 5 showed low percentages (less than 30%). Among the 3 oligodendroglial tumors, only 1 case showed positive for 1p36 loss, on the other hand, three cases out of 6 pure astrocytic tumors showed positive for 1p36. There was no significant difference between 1p loss-positive group and 1p loss–negative group with respect to PFS or OS (Figure S2). In order to compare the prognostic significance between 1p loss status and oligodendroglial component, we next divided these 9 patients into oligodendroglial tumors and pure astrocytic tumors and examine the PFS and OS. There was no significant difference between oligodendroglial tumors and pure astrocytic tumors in PFS and OS, however, the each survival curves of oligodendroglial tumors showed distinctly better prognosis than pure astrocytic tumors.

## Discussion

Because the favorable prognosis of oligodendroglial tumor including AO and AOA has been clinically recognized [1], , the proper pathological diagnosis for these tumors is required. In fact, the recent clinical Phase III trial of anaplastic gliomas revealed that AO and AOA shared the similar prognosis, which was better than that for AA [14]. Regarding GBMO, although its prognostic evaluation still remains controversial [5], [6], [7], [8], [9], [10], here we have shown that the prognosis of GBMO, at least in terms of PFS, but also in terms of the tendency for OS, was significantly better than that of GBM. In this study, we have performed the alternative categorization of high-grade gliomas throughout Grade III and IV, i.e., into oligodendroglial tumor (AO, AOA and GBMO) and pure astrocytic tumor (AA and GBM), and obtained the notable result of the survival analysis (Fig. 2c and 3c). The survival curves of OS and PFS of the two groups were almost similar to, or much more significant than, that of the conventional categorization into Grade III and IV (Fig. 2a and 3a). Furthermore, the survival analysis within the group of pure astrocytic tumors, more specifically pure astrocytic high grade gliomas, exhibited the unexpected conclusion that the prognosis of AA and GBM was almost identical in OS or even PFS (Fig. 2d and 3d). Based on these results, we concluded that the presence of histological oligodendroglial tumor component, purely or even partially, is a critical prognostic factor for high-grade glioma throughout Grade III and IV, although additional studies to increase the number of the cases for AA (n = 13) which was rather less than that of GBM (n = 57) might be required for further confirmation.

In our histological review process, we defined the oligodendroglial tumor component by identification of the groups of the cells with an obvious perinuclear halo (fried egg appearance) in H&E section; however we did not set the definite numeric value for the proportion of the oligodendroglial tumor component. Establishing the definite criteria for the proportion of oligodendroglial tumor component for diagnosis can be difficult, because the pathological materials obtained by biopsy or even total resection usually reflect the partial aspect of the lesion; in fact, it varied between 10 to 25% in a previous report [1]. The reason that the percentage of GBMO in our series (25%) was higher than previous report (5–20%) [6], [7], [9] might be explained by such difference of diagnostic criteria for the oligodendroglial tumor component. To distinguish the oligodendroglial tumor from the astrocytic tumor, the immunohistochemical specific marker has not been identified, while the detection of loss of chromosome 1 (1p) and chromosome 19 (19q) by FISH is established [11], [15], [16]. However, because the consensus diagnostic criteria of the proportion of cells with 1p and 19q deletion has not been built yet, previous reports indicated the variable cut-off values [17], [18], [19], and furthermore, FISH technique has not always been one of the routine clinical examinations in general hospitals, and the aged, long term-fixed pathological specimens are sometimes not suitable for this analysis. In fact, we failed the FISH analysis in 9 out of 18 cases because of the poor preservation state and/or the shortage of the specimen. Interestingly, four cases which represented positive for 1p loss included 3 GBMs without histological oligodendroglial tumor component. Moreover, the survival analysis for these 9 cases revealed unexpected results that the histological evaluation for oligodendroglial tumor component was more sensitive factor rather than the FISH analysis for 1p-loss (Figure S2), although it was not statistically significant due to small number of the cases. These results also suggest the diagnostic significance of histological evaluation for oligodendroglial tumor component. In addition, the histological oligodendroglial features including perinuclear halo (classical histology) was noted as a strong predictor of clinical outcome, rather than 1p/19q status [20]. Hence, the fact that the histological identification of the cells with an obvious perinuclear halo (fried egg appearance) in H&E section is enough to discuss the prognosis, as we presented here, is quite important for the majority of the pathologists to make a routine diagnosis and the neurosurgeons to treat the patients with high-grade glioma.

A critical question has arisen: how does the presence of oligodendroglial tumor component, even partially, yield to the favorable prognosis? One of the possible hypotheses is that the cell biology of oligodendroglial tumor would differ from that of astrocytic tumor. The therapeutic sensitivity of 1p/19q-loss oligodendroglioma to chemotherapy and radiation was discussed previously, although it is not clear whether oligodendroglioma represent a tumor type that is more responsive to cytotoxic therapies or whether these tumors are more biologically indolent [15]. The experimental study using oligodendroglial tumor cell line would be expected to answer this query, although there are currently no available cell lines derived from human oligodendroglial tumor.

In conclusion, we emphasize the prognostic significance to identify the oligodendroglial tumor component, even partially, in routine H & E sections of the high-grade gliomas, and would propose the alternative histological grading system of Grade III including GBMO as well as AO and AOA.

## Supporting Information

Figure S1 **The histological appearance of typical AA (a), AO (b), AOA (c) and GBM (d).** a: AA is composed of astrocytic cells with moderate atypia. There is no evident necrosis, prominent vascular proliferation, or oligodendroglial tumor component. b: AO is composed of oligodendrocytic cells with obvious perinuclear halo. c: In AOA, astrocytic cells are intermingled with oligodendrocytic cells. There is no evident necrosis. d: In GBM, diffuse infiltration of pleomorphic tumor cells is observed and the microvascular proliferation is prominent. The foci of necrosis are found in other fields. (The scale bars represent 50 micrometers.).
(TIF)

Figure S2 **Survival analysis based on 1p loss status or histological subclassification.** The graph shows comparison of progression-free survival (PFS) or overall survival (OS) according to 1p loss status (a, b) and histological subclassification (c, d). Although any of them shows no statistical siginificance between them, oligodendroglial tumor is associated with longer survival.
(TIF)

Figure S3 **The overlayed survival curves.** The survival curves of the Grade III and oligodendroglial tumor (AO, AOA, GBMO; oligo), and Grade IV and pure astrocytic tumor (AA, GBM; pure astro) were almost identical, respectively.
(TIF)

Table S1 **The result of FISH analysis for 1p36 loss.** AOA: anaplastic oligoastrocytoma, AA: anaplastic astrocytoma, GBM: glioblastoma, GTR: gross total resection, PR: partial resection, STR: subtotal resection, TMZ: Temozolomide, ACNU: Nimustine hydrochloride, CR: complete response, SD: stable disease, NA: not available, D: death, PFS: progression free survival, OS: overall survival.
(DOCX)
