# Supplementary figures and images for: Prognostic Implication of Histological Oligodendroglial Tumor Component: Clinicopathological Analysis of 111 Cases of Malignant Gliomas
Source: PLoS One. 2012 Jul 24;7(7):e41669. doi: 10.1371/journal.pone.0041669 (PMC3404002; doi:10.1371/journal.pone.0041669)

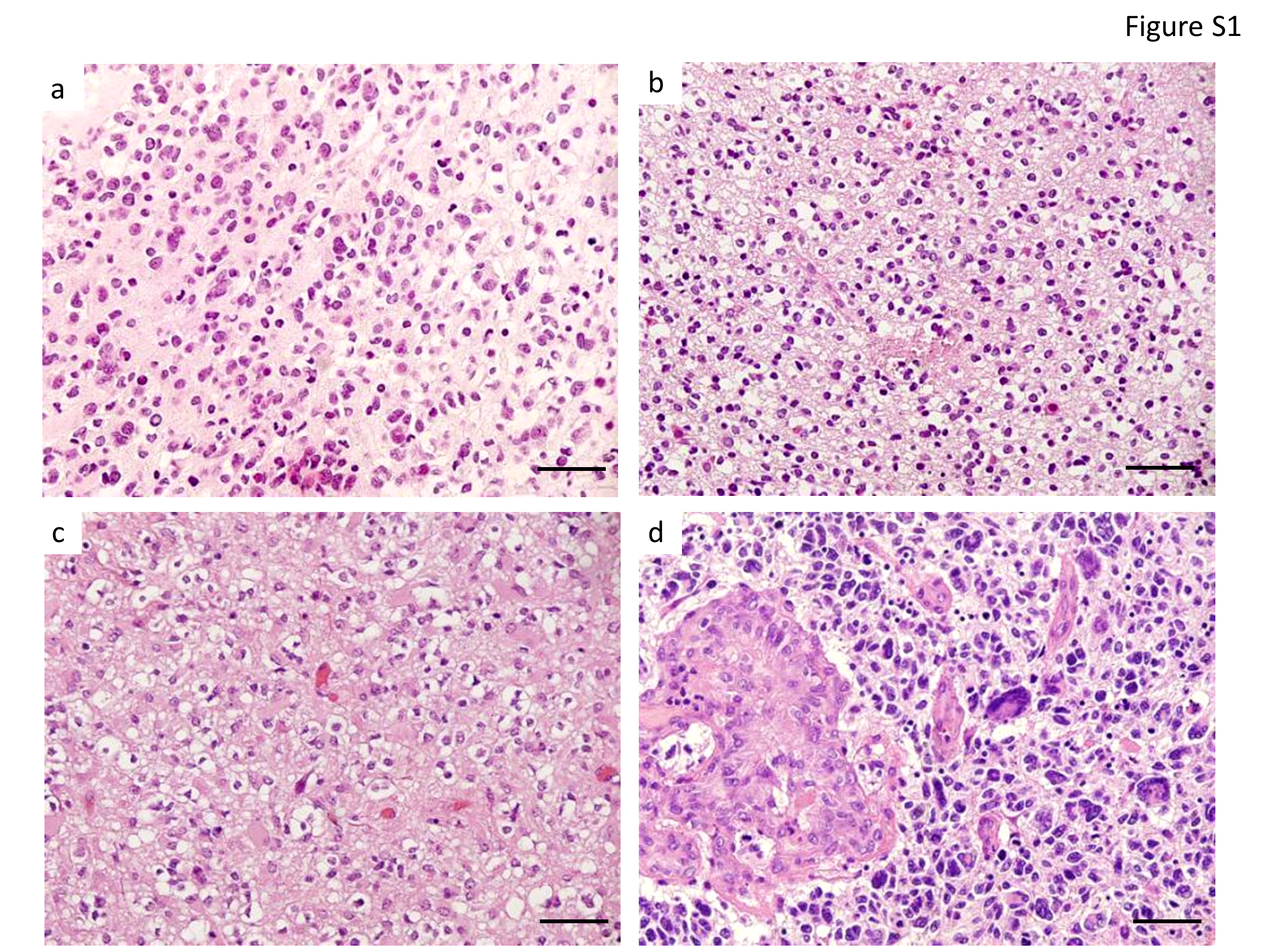

Supplement: Figure S1 — The histological appearance of typical AA (a), AO (b), AOA (c) and GBM (d). a: AA is composed of astrocytic cells with moderate atypia. There is no evident necrosis, prominent vascular proliferation, or oligodendroglial tumor component. b: AO is composed of oligodendrocytic cells with obvious perinuclear halo. c: In AOA, astrocytic cells are intermingled with oligodendrocytic cells. There is no evident necrosis. d: In GBM, diffuse infiltration of pleomorphic tumor cells is observed and the microvascular proliferation is prominent. The foci of necrosis are found in other fields. (The scale bars represent 50 micrometers.). (TIF) [file pone.0041669.s001.tif]

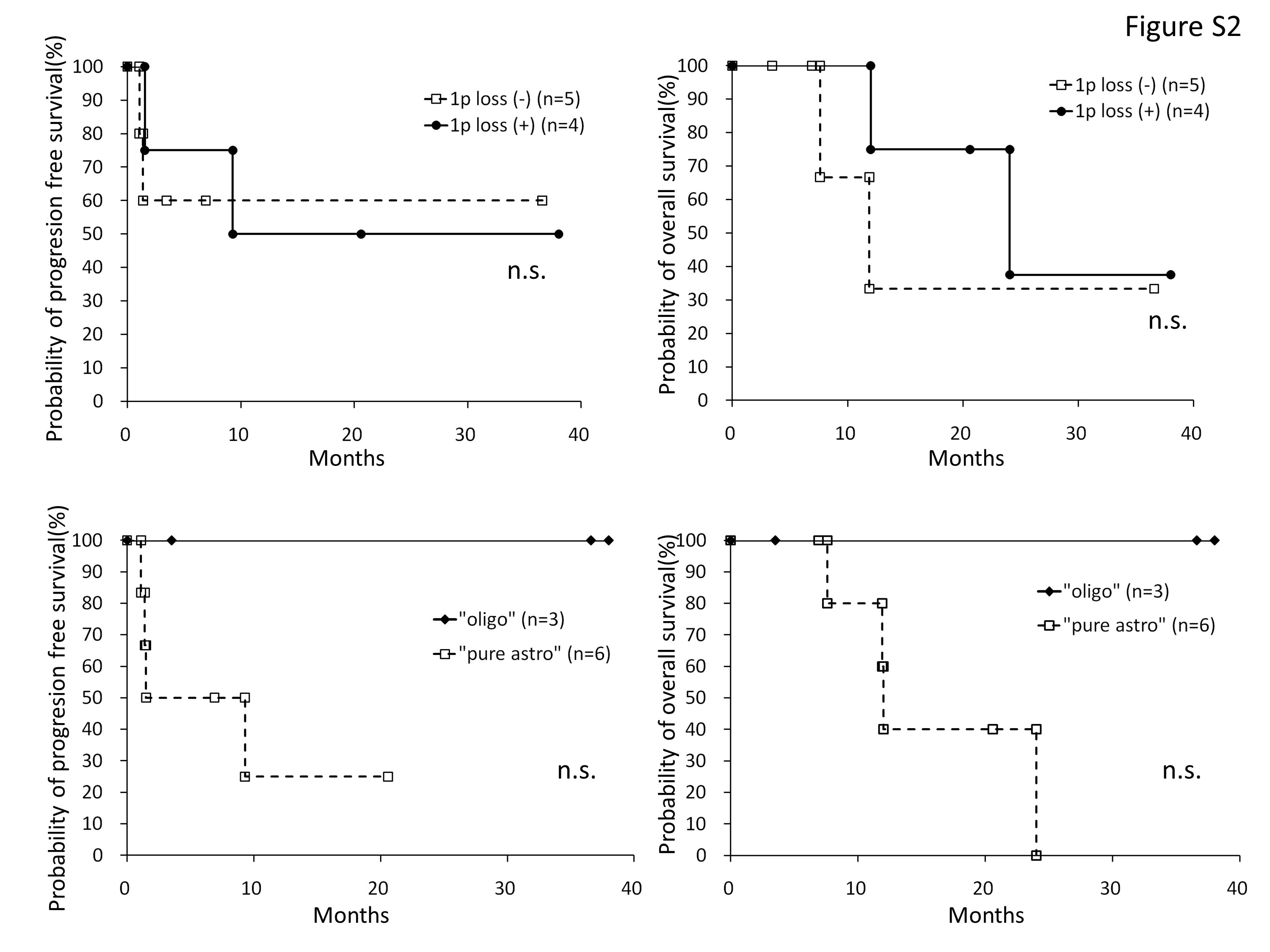

Supplement: Figure S2 — Survival analysis based on 1p loss status or histological subclassification. The graph shows comparison of progression-free survival (PFS) or overall survival (OS) according to 1p loss status (a, b) and histological subclassification (c, d). Although any of them shows no statistical siginificance between them, oligodendroglial tumor is associated with longer survival. (TIF) [file pone.0041669.s002.tif]

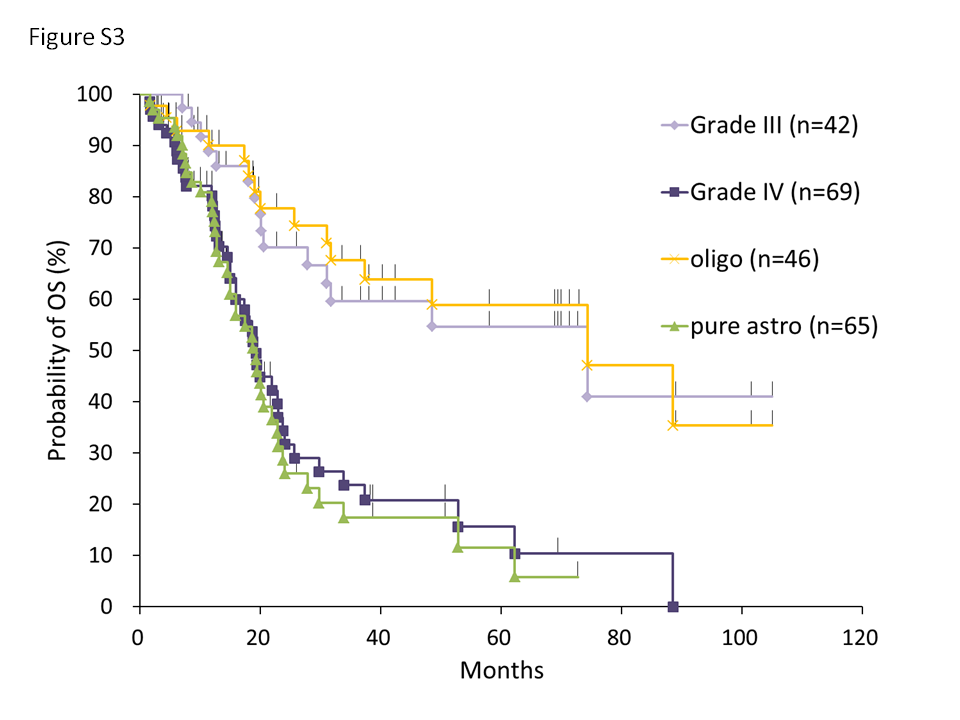

Supplement: Figure S3 — The overlayed survival curves. The survival curves of the Grade III and oligodendroglial tumor (AO, AOA, GBMO; oligo), and Grade IV and pure astrocytic tumor (AA, GBM; pure astro) were almost identical, respectively. (TIF) [file pone.0041669.s003.tif]
