# Supplementary material for: Prognostic Implication of Histological Oligodendroglial Tumor Component: Clinicopathological Analysis of 111 Cases of Malignant Gliomas
Source: PLoS One. 2012 Jul 24;7(7):e41669. doi: 10.1371/journal.pone.0041669 (PMC3404002; doi:10.1371/journal.pone.0041669)
Supplement: Table S1 — The result of FISH analysis for 1p36 loss. AOA: anaplastic oligoastrocytoma, AA: anaplastic astrocytoma, GBM: glioblastoma, GTR: gross total resection, PR: partial resection, STR: subtotal resection, TMZ: Temozolomide, ACNU: Nimustine hydrochloride, CR: complete response, SD: stable disease, NA: not available, D: death, PFS: progression free survival, OS: overall survival. (DOCX) [file pone.0041669.s004.docx]

**Table S1. The result of FISH analysis for 1p36 loss**

| **Case**  **No.** | **Age** | **Sex** | **Dx.** | **Surgery** | **Chemo**  **-therapy** | **Radiation** | **Follow-up** | **1p36 loss**  **(%)** | **1p loss** |
| --- | --- | --- | --- | --- | --- | --- | --- | --- | --- |
| 1 | 70 | M | AOA | GTR | TMZ | 54Gy/27Fr | No recurrence,  alive at 3.5 month | 9.8 | - |
| 2 | 58. | M | AOA | GTR | TMZ | 54Gy/27fr | No recurrence,  alive at 36.6 month | 14.9 | - |
| 3 | 50 | F | AOA | GTR | TMZ | 54Gy/27f | No recurrence,  alive at 38.0 month | 54.8 | + |
| 4 | 33 | F | AA | PR | TMZ | none | Lost to follow-up  at 6.9 month | 27 | - |
| 5 | 67 | M | GBM | STR | TMZ | 60Gy/30f | Progression at 1.4 month  death at 11.9 month | 22.6 | - |
| 6 | 76 | F | GBM | STR | none | 22Gy/11f | Progression at 1.1 month  death at 7.6 month | 24.1 | - |
| 7 | 73 | M | GBM | STR | TMZ | 60Gy/30Fr | No recurrence,  alive at 20.6 month | 45.7 | + |
| 8 | 73 | F | GBM | GTR | ACNU | 60Gy/30f | Progression at 1.5 month  death at 24.0 month | 46.4 | + |
| 9 | 65 | M | GBM | STR | TMZ | 60Gy/30fr | Progression at 9.3 month  death at 12.0 month | 50.8 | + |

AOA: anaplastic oligoastrocytoma, AA: anaplastic astrocytoma, GBM: glioblastoma,

GTR: gross total resection, PR: partial resection, STR: subtotal resection, TMZ: Temozolomide, ACNU: Nimustine hydrochloride, CR: complete response, SD: stable disease, NA: not available, D: death, PFS: progression free survival, OS: overall survival.
